# Diagnosis of streptococcal pharyngotonsillitis in children and adolescents: clinical picture limitations☆

**DOI:** 10.1016/j.rpped.2014.04.001

**Published:** 2014-12

**Authors:** Aurelino Rocha Barbosa, Cláudia Di Lorenzo Oliveira, Maria Jussara Fernandes Fontes, Laura Maria de Lima Bezário Facury Lasmar, Paulo Augusto Moreira Camargos

**Affiliations:** a Universidade Federal de São João del-Rei (UFSJ), Divinópolis, MG, Brazil; b Universidade Federal de Minas Gerais (UFMG), Belo Horizonte, MG, Brazil

**Keywords:** Pharyngitis, Diagnosis, Streptococcus, Children, Adolescent

## Abstract

**OBJECTIVE:**

To assess the utility of clinical features for diagnosis of streptococcal pharyngotonsillitis in pediatrics.

**METHODS:**

A total of 335 children aged 1-18 years old and presenting clinical manifestations of acute pharyngotonsillitis (APT) were subjected to clinical interviews, physical examinations, and throat swab specimen collection to perform cultures and latex particle agglutination tests (LPATs) for group A streptococcus (GAS) detection. Signs and symptoms of patients were compared to their throat cultures and LPATs results. A clinical score was designed based on the multivariate logistic regression analysis and also was compared to throat cultures and LPATs results. Positive throat cultures and/or LPATs results were used as a reference standard to establish definitive streptococcal APT diagnosis.

**RESULTS:**

78 children (23.4%) showed positivity for GAS in at least one of the two diagnostic tests. Coryza absence (odds ratio [OR]=1.80; *p*=0.040), conjunctivitis absence (OR=2.47; *p*=0.029), pharyngeal erythema (OR=3.99; *p*=0.006), pharyngeal exudate (OR=2.02; *p*=0.011), and tonsillar swelling (OR=2.60; *p*=0.007) were significantly associated with streptococcal pharyngotonsilitis. The highest clinical score, characterized by coryza absense, pharyngeal exudate, and pharyngeal erythema had a 45.6% sensitivity, a 74.5% especificity, and a likelihood ratio of 1.79 for streptococcal pharyngotonsilitis.

**CONCLUSIONS:**

Clinical presentation should not be used to confirm streptococcal pharyngotonsilitis, because its performance as a diagnostic test is low. Thus, it is necessary to enhance laboratory test availability, especially of LPATs that allow an acurate and fast diagnosis of streptococcal pharyngotonsilitis.

## Introduction

Acute pharyngotonsillitis (APT) is a common health problem worldwide, especially in children, which is most often related to benign viral and self-limiting infections. However, a non-negligible number of these infections are of bacterial etiology, and in this case, the β-hemolytic group A streptococcus (GAS) is the main causative agent, which can lead to severe complications, with great individual, collective, social, and economic impact; the main complication is rheumatic fever (RF).^1^

RF is a non-suppurative complication of APT caused by GAS and is characterized by the appearance of inflammatory changes in the joints, skin, heart, and central nervous system, disclosing different combinations and degrees of severity. Of these, rheumatic carditis (RC) is the most feared disease manifestation, as it is the only one that can result in sequelae, often severe, and lead to death.^2^

Considering its possible complications, it is essential to attain a correct diagnosis and adequate management of streptococcal APT, as its timely treatment (up to nine days of symptom onset) is effective in preventing both suppurative and non-suppurative complications.^3^ The diagnosis is challenging, as studies show a large overlap between the clinical presentation of viral and streptococcal APT, with no clinical feature that, individually, can confirm or rule out the diagnosis of streptococcal APT.^4^

However, there is no consensus of uniformity regarding the diagnosis and management of APT^5^ and some authors have developed scores to classify the risk of streptococcal APT, with varying results.^6^^-^ 9 Additionally, diagnostic laboratory tests, namely, culture or rapid antigen detection testing (RADT) are not always readily available or are not part of the reality of professionals that work directly with patients with APT.^10^

According to estimates by the World Health Organization, approximately 600 million new cases of symptomatic APT caused by GAS occur annually in children worldwide. Of these, about 500,000 develop RF, and approximately develop 300,000 RC. Most of these cases occur in less developed countries, with three times higher prevalence of RF in these countries, including Latin America, than in developed countries.^1^

In Brazil, the Brazilian Societies of Cardiology, Pediatrics, and Rheumatology estimates that approximately 10 million cases of streptococcal APT occur every year, which would account for 30,000 new cases of RF, of which approximately 15,000 would develop cardiac involvement. In 2007 alone, the Brazilian Unified Health System (Sistema Único de Saúde SUS]) spent approximately R$ 170 million in hospital admissions due to RF or RC and, of cardiac surgeries performed that year, 31% occurred in patients with RF sequelae.^2^

The aim of this study is to evaluate the usefulness of the clinical picture for the diagnosis of streptococcal APT and to define a group of signs and symptoms that can maximize their diagnostic power. 

## Method

This study was conducted in patients aged 18 or younger that presented with complaints of sore throat and/or pharyngeal and/or tonsillar erythema at admission in an emergency department and a pediatric outpatient clinic in the city of Belo Horizonte, state of Minas Gerais, Brazil, and was approved by the Ethics Committee of Universidade Federal de São João Del Rey (UFSJ) under number 05705112.6.0000.5545. Patients who had received benzathine penicillin in the past 30 days or any other antimicrobial agent in the past 15 days were excluded from the study.

Signs and symptoms associated with upper respiratory tract and APT were assessed and recorded on a standardized form. Additionally, signs and symptoms that were often not assessed in similar studies were investigated, such as the following: gingivitis, tearing, tonsillar pillar erythema, sneezing, and prostration during and between fever peaks (>38.5°C):

After obtaining consent from the parents or guardians, the physical examination was performed, and the latex particle agglutination test (LPAT) and oropharyngeal cultures obtained independently and in a blinded fashion were performed. Two swabs were obtained from each patient, one for the LPAT (PathoDx^(r)^, DPC - Los Angeles, United States) and the other for the conventional culture on sheep blood agar plate at 5%. 

Differently from almost all published studies on this subject, the protocol included not only the throat culture as the gold standard, but rather a better and more robust reference standard, that is, the combination in parallel of oropharyngeal culture results and LPAT. The definitive diagnosis of streptococcal APT was given when the culture or LPAT were positive; conversely, a negative result for both culture and LPAT ruled out the diagnosis of streptococcal APT, thereby increasing sensitivity.

Sensitivity and specificity were calculated for each sign and symptom, with their respective 95% confidence intervals (95% CI). The positive likelihood ratio (LR+), odds ratio (OR), and *p*-value for each sign and symptom were also calculated. The chi-squared test was used to determine statistical significance. 

Signs and symptoms that showed *p*-values ​​<0.20 were submitted to multivariate analysis using a logistic regression model to develop a clinical prediction score. The sensitivity, specificity, and LR+ were then calculated for each score.

## Results

A total of 335 patients were considered eligible, of whom 56 (16.72%) were positive for GAS culture, 71 (21.2%) for LPAT, and 78 (23.4%) for LPAT and/or culture. The agreement between the tests was 91.3%, with a kappa of 0.72. The sensitivity, specificity, LR+ and the OR and *p*-values ​​were calculated for each clinical feature observed in the interview and clinical examination, and are shown in Table 1. 

**Table 1:**
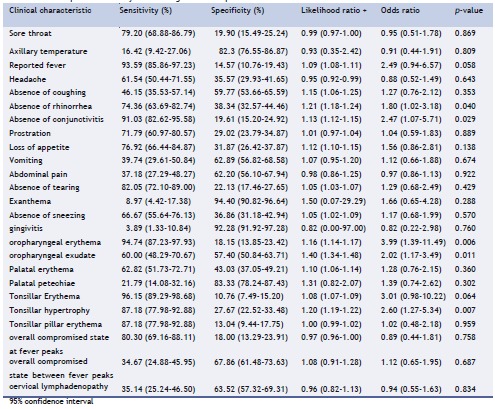
C Clinical picture accuracy for the diagnosis of streptococcal APT.

It can be observed that the clinical characteristic with the highest sensitivity for the diagnosis of APT by GAS was tonsillar erythema (96.2%), followed by oropharyngeal erythema (94.7%), fever reported by the parent or guardian (93.6%), and absence of conjunctivitis (91.0%). However, all these characteristics had low specificity (10.8%, 18.2%, 14.6%, and 19.6%, respectively). The clinical characteristic with the highest specificity was the presence of exanthema (94.4%), followed by gingivitis (92.3%), palatal petechiae (83.3%), and axillary temperature measured at the consultation >38.5ºC (82.3%), but all with low sensitivity (8.9%, 3.9%, 21.8%, and 16.4%, respectively). 

The characteristics statistically associated with APT by GAS (*p*<0.05) were absence of rhinorrhea (OR=1.80, *p*=0.040); absence of conjunctivitis (OR=2.47, *p*=0.029); oropharyngeal erythema (OR=3.99, *p*=0.006); oropharyngeal exudate (OR=2.02, *p*=0.011); and tonsillar hypertrophy (OR=2.60, *p*=0.007).

The characteristics with the highest likelihood ratios were exanthema (1.50); oropharyngeal exudate (1.40), and palatal petechiae (1.31). However, both palatal petechiae and exanthema had wide confidence intervals.

The characteristics included in the logistic regression analysis were: reported fever, absence of rhinorrhea, absence of conjunctivitis, loss of appetite, oropharyngeal erythema, oropharyngeal exudate, tonsillar erythema, and hypertrophy. The analysis was performed using the stepwise model. The following characteristics were retained in the model, according to the program: absence of rhinorrhea; oropharyngeal erythema, and exudate (Table 2). The results of the combination of these characteristics generated a score in which each corresponds to a point, as shown in Table 3.

**Table 2:**
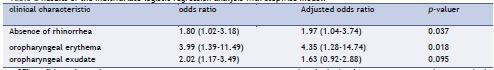
Results of the multivariate logistic regression analysis with stepwise model.

**Table 3:**
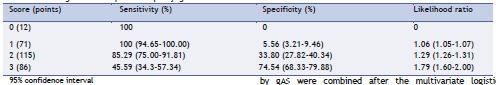
Score and performance of clinical score, consisting of absence of rhinorrhea, oropharyngeal erythema, and exudate for the clinical diagnosis of streptococcal pharyngotonsillitis.

As expected, it can be observed that the higher the score, the greater the specificity and the lower the sensitivity of the clinical picture. Thus, a score of 3 points, or the presence of the three characteristics (absence of rhinorrhea, oropharyngeal exudate, and oropharyngeal erythema) corresponds to a sensitivity of 45.6%, a specificity of 74.5%, and an LR+ of 1.79. This LR+ is higher than in any of the clinical characteristics alone, corresponding to the best performance of the clinical picture in these results.

## Discussion

In this study, the prevalence of GAS in children and adolescents with APT was 23%, lower than that estimated by the work of Shaikh, Leonard, and Martin.^11^ In their meta-analysis, the prevalence of the 14 studies analyzed together was 37%. However, the authors emphasize that there was great heterogeneity among these studies and that prevalence ranged from 17% to 58% in them.

Several studies have been published aiming to elucidate the clinical picture accuracy for the etiological diagnosis of APT in children, with variable results.^7^^,^ 8 ^,^ 12 ^-^ 15 Rimoin *et al* 16 demonstrated the existence of significant variation in the clinical presentation of APT in four different countries, suggesting it is inadequate to extrapolate conclusions about the clinical picture accuracy from one region to another. Therefore, the importance of the present study is emphasized, conducted in a country where RF and its complications are a major public health problem. 

Clinical characteristics statistically associated with the presence of GAS vary among studies:^7^^,^ 8 ^,^ 12 ^-^ 15 palatal petechiae; anterior cervical adenopathy; oropharyngeal exudate; sore throat; tonsillar hypertrophy; scarlet fever-like rash, fever >38°C; muscle pain; bad breath; oral ulcers; gastrointestinal symptoms; contact with patients with APT by GAS; age 5 to 12 years; and oropharyngeal erythema appear alone or in different combinations in the several studies. Rhinorrhea, cough, and conjunctivitis usually appear as symptoms associated with viral infection (*p*<0.05). However, none of these characteristics is unique to streptococcal or viral APT. Moreover, none of them show, concomitantly, high sensitivity and specificity for the diagnosis of streptococcal APT. 

The results of the present study support this information, showing characteristics with high sensitivity, but low specificity (tonsillar erythema, oropharyngeal erythema, reported fever, and absence of conjunctivitis) and conversely, characteristics with high specificity but low sensitivity (presence of exanthema, gingivitis, palatal petechiae, and axillary temperature >38.5°C). The characteristic with the greatest balance between sensitivity and specificity was oropharyngeal exudate, with 60% and 57.4%, respectively.

Furthermore, the characteristics statistically associated with the presence of GAS in this study, namely, the absence of rhinorrhea, absence of conjunctivitis, oropharyngeal erythema, oropharyngeal exudate, and tonsillar hypertrophy, were different from those obtained in other studies performed in Brazil.^8^^,^ 15 ^,^ 16 This further supports the finding of the variability of manifestations in streptococcal APT, even within the same country. 

None of the characteristics analyzed in this study showed a high likelihood ratio. The characteristics with higher likelihood ratios were: exanthema (LR=1.50, 95% CI: 0.07-29.29); oropharyngeal exudate (LR=1.40, 95% CI: 1.34-1.48); and palatal petechiae (LR=1.31, 95% CI: 0.82-2.07). It was observed, however, that both exanthema and palatal petechiae showed wide confidence intervals that include 1, which makes the results inaccurate and of little significance. Nevertheless, any of these values cause very little change to ​​the pre-test probability of streptococcal APT. Considering a mean prevalence of 30% of GAS as the cause of APT,^17^ a positive likelihood ratio of 1.4, as observed for oropharyngeal exudate, would increase the post-test likelihood to only about 35%.

In an attempt to increase the diagnostic usefulness of the clinical picture, some authors have proposed the use of clinical prediction scores. Thus, McIsaac *et al* 6 obtained a score with a sensitivity and specificity of 66.1% and 85.4%, respectively. However, the sample used by these authors consists mostly of adult patients, although their score is corrected for age.

Smeesters *et al* 8 and Joachim, Campos and Smeesters^9^ developed their scores aiming to diagnose cases of non-streptococcal APT. They obtained scores with sensitivity and specificity for this purpose of 41% and 84%, and 35% and 88%, respectively. Nonetheless, the aim of these scores is to provide an alternative to microbiological tests for the correct management of the APTs, that is, to provide adequate antibiotic therapy when required and avoid its use when unnecessary, especially given the current concern with bacterial resistance development.^18^

In the present study, a sensitivity of 45.6% was obtained (95% CI: 34.3 to 57.3%) and a specificity of 74.5% (95% CI: 68.3 to 79.9%) when the three associated symptoms of APT by GAS were combined after the multivariate logistic regression. This also corresponds to a likelihood ratio of 1.79 (95% CI: 1.60-2.00). As the score has low sensitivity, it does not allow ruling out the diagnosis of streptococcal APT only by this criterion. Despite the moderate specificity, the score of 3 points has a likelihood ratio that is considered low. Once again, with the example of a streptococcal APT prevalence of 30% (pre-test likelihood), a post-test likelihood of 40% would be obtained, which is not enough to confirm the diagnosis.

Considering the important impact of APT, especially RF and RC, particularly in less developed countries, it is necessary to consider and define what would be the best strategy for the diagnosis and management of APTs, taking into account individual and collective risks, availability of laboratory tests for the diagnosis, the limitations of these tests, and the costs of each strategy, without losing sight of the possible complications of each one.

Thus, some authors have made efforts to define which strategy would be the most appropriate for APT management.^19^^-^ 22 Giraldez-Garcia *et al*,^19^ for instance, analyzed the cost, the effectiveness, and the cost/effectiveness ratio of six different strategies for APT management in Spain and concluded that the best strategy for that country, in terms of cost and cost/effectiveness, would be the use of a clinical score, followed by rapid test for patients with a high score, for diagnostic confirmation. 

By applying this strategy to the present study population, 30% of the patients would be tested, an inadequate antibiotic would not be used in any of the cases negative for GAS, but 57% of positive cases would not be properly treated, an unacceptable percentage. Nevertheless, this extrapolation is only a theoretical exercise, as the information used by these researchers may not apply to Brazil, in the context of the abovementioned considerations on the variability of APT between different regions. Moreover, the clinical criteria used by the Spanish authors differ from those found in this study.

The literature review showed only one study that, as the present, used the combination of culture and rapid antigen detection test as the reference standard.^9^ However, in that study, children undergoing culture were not submitted to RADT and *vice versa*, as the study was performed in two steps, and one of the tests was used in each step. In the present, all patients were submitted to the two tests. Considering that, in practice, the positive result of both the culture and the RADT should be seen as positive, it is considered that the methodology applied in this study is closer to the reality. Furthermore, the use of two highly specific tests reduces the possibility of false negative results, by increasing the standard sensitivity. 

This study has some limitations. First, due to the incapacity of reference standards, LPAT, culture, or a combination of both, to differentiate patients with GAS, one may have underestimated the actual accuracy of the clinical picture for the diagnosis for APT. Some studies estimate a prevalence of approximately 10% of GAS in healthy carriers;^11^^,^ 23 however, there are no accurate and practical methods to differentiate symptomatic patients (infected by another microorganism rather than GAS) from those infected by it. Moreover, as the study had a cross-sectional design, the impact that subsequent assessments might have on clinical picture accuracy could not be assessed. That would be important, as a frequent conduct in APT cases is to wait for the evolution within 24 to 48 hours to reassess children and define the therapeutic strategy.

No studies in the literature that used such approach were found. Thus, it would be important, given the information already available and the contribution of this work, to perform longitudinal studies to sequentially evaluate patients and assess whether such a measure would increase the diagnostic accuracy of the clinical picture. Moreover, it would be worthwhile to perform a cost/effectiveness analysis that would more adequately apply to the Brazilian reality, and that of other developing and underdeveloped countries. 

The present results show that the clinical picture should not be used alone to confirm the episode of streptococcal APT. Even when some clinical features are combined, the resulting positive likelihood ratio does not allow increasing the post-test likelihood to a sufficiently high value to confirm the diagnosis of streptococcal APT. It is necessary to increase the availability of confirmatory laboratory tests, especially RADT, which allows a rapid and accurate diagnosis of the streptococcal APT episode.

In view of the Brazilian reality, where there is scarce availability and request for confirmatory laboratory tests in a continuous feedback, it would be important that medical societies, such as the Brazilian Society of Pediatrics, the Brazilian Society of Infectious Diseases, and the Brazilian Society of Cardiology, as well as the Ministry of Health, issue a recommendation for the correct diagnosis and management of APT. Thus, there would be an incentive, to medical professionals, as well as laboratories, to request and provide the necessary tests for the diagnosis and management of APT. 
