# Supplementary material for: Diagnosis of streptococcal pharyngotonsillitis in children and adolescents: clinical picture limitations
Source: Rev Paul Pediatr. 2014 Dec;32(4):285–91. doi: 10.1016/j.rpped.2014.04.001 (PMC4311780; doi:10.1016/j.rpped.2014.04.001)
Supplement: Translation

# Translation: Diagnóstico da faringoamigdalite estreptocócica em crianças e adolescentes: limitações do quadro clínico☆

**Authors:** Aurelino Rocha Barbosa, Cláudia Di Lorenzo Oliveira, Maria Jussara Fernandes Fontes, Laura Maria de Lima Bezário Facury Lasmar, Paulo Augusto Moreira Camargos

**Affiliations:** a Universidade Federal de São João del-Rei (UFSJ), Divinópolis, MG, Brasil; ı Universidade Federal de Minas Gerais (UFMG), Belo Horizonte, MG, Brasil

**Keywords:** Faringite, Diagnóstico, Streptococcus, Criança, Adolescente

## Abstract

**OBJETIVO:**

Avaliar a utilidade do quadro clínico para o diagnóstico da faringoamigdalite estreptocócica na faixa pediátrica.

**MÉTODOS:**

335 indivíduos de 1 a 18 anos com quadro clínico de faringoamigdalite foram submetidos a anamnese, exame clínico, cultura e teste de aglutinação de partículas do látex (TAPL) para o estreptoco β-hemolítico do grupo A (EBHGA) em *swab* orofaríngeo. Os sinais e sintomas foram comparados ao resultado da cultura e do TAPL e, em seguida, também o foi o agrupamento de sinais e sintomas definido por regressão logística multivariada, utilizado para gerar um escore clínico. Para o diagnóstico definitivo de faringoamigdalite estreptocócica, o padrão de referência foi a cultura e o TAPL, em paralelo.

**RESULTADOS:**

78 indivíduos (23,4%) apresentaram resultados positivos para EBHGA em pelo menos um dos testes. Ausência de coriza (OR=1,80; *p*=0,04); ausência de conjuntivite (OR=2,44; *p*=0,029); hiperemia de orofaringe (OR=3,99; *p*=0,006); exsudato de orofaringe (OR=2,02; *p*=0,011) e hipertrofia de amígdalas (OR=2,60; *p*=0,007) apresentaram associação significante com a faringoamigdalite estreptocócica. A pontuação máxima no escore clínico, no qual figuram três características (ausência de coriza, exsudato de orofaringe e hiperemia de orofaringe) correspondeu a uma sensibilidade de 45,6%, uma especificidade de 74,5% e uma *likelihood ratio* de 1,79 para a presença de faringoamigdalite estreptocócica.

**CONCLUSÃO:**

O quadro clínico não deve ser usado isoladamente para confirmar o episódio de faringoamigdalite estreptocócica por apresentar um baixo desempenho diagnóstico. É necessário aumentar a disponibilidade de testes laboratoriais, em especial o TALP, que permite o diagnóstico rápido e acurado deste episódio.

## Introdução

A faringoamigdalite aguda (FAA) é um problema de saúde frequente em todo o mundo, especialmente entre as crianças, estando na maioria das vezes relacionada a infecções virais benignas e auto limitadas. Contudo, um número não desprezível dessas infecções é de etiologia bacteriana e, neste caso, o estreptococo β-hemolítico do grupo A (EBHGA) é o seu principal agente causal, podendo levar a complicações graves e de grande impacto individual e coletivo, social e econômico, das quais a principal é a febre reumática (FR).^1^

A febre reumática é uma complicação não supurativa das FAA causadas por EBHGA e se caracteriza pelo surgimento de alterações inflamatórias nas articulações, pele, coração e sistema nervoso central, em combinações e de gravidade variáveis. Dessas, a cardite reumática (CR) é a manifestação mais temida da doença, uma vez que é a única que pode deixar sequelas, muitas vezes graves, e conduzir ao óbito.^2^

Em vista das suas possíveis complicações, torna-se fundamental o correto diagnóstico e manejo das FAA estreptocócicas, uma vez que o seu tratamento a tempo (até 9 dias do início dos sintomas) é efetivo em prevenir estas complicações, supurativas e não supurativas.^3^ O diagnóstico é um desafio, uma vez que os estudos demonstram larga sobreposição entre os quadros clínicos de FAA virais e estreptocócicas, não havendo uma característica clínica que, individualmente, possa confirmar ou descartar o diagnóstico de FAA estreptocócica.^4^

Entretanto, não existe uniformidade entre os consensos para o diagnóstico e manejo das FAA^5^ e alguns autores vêm desenvolvendo escores para classificar o risco de FAA estreptocócica, com resultados variáveis.^6^^-^ 9 Além disso, nem sempre os exames laboratoriais diagnósticos, quais sejam, cultura ou teste rápido de identificação de antígenos (TRIA), estão prontamente disponíveis ou não fazem parte da realidade dos profissionais que lidam diretamente com os pacientes que apresentam FAA.^10^

Segundo estimativas da Organização Mundial da Saúde ocorrem, anualmente, cerca 600 milhões de novos casos de FAA por EBHGA sintomática entre crianças no mundo todo. Desses, cerca de 500 mil podem evoluir com FR e cerca de 300 mil com CR. A maior parte desses casos acontece em países menos desenvolvidos, sendo a prevalência de FR nesses países, incluindo a América Latina, três vezes maior que nos países mais desenvolvidos.^1^

No Brasil, as Sociedades Brasileiras de Cardiologia, Pediatria e Reumatologia estimam que, anualmente, ocorram cerca de 10 milhões de FAA estreptocócicas, que seriam responsáveis por 30 mil novos casos de FR, dos quais aproximadamente 15 mil evoluiriam com acometimento cardíaco. Somente em 2007 foram gastos pelo Sistema Único de Saúde (SUS) cerca de 170 milhões de reais em internações decorrentes de FR ou CR, sendo que, das cirurgias cardíacas realizadas nesse ano, 31% ocorreram em pacientes com seqüelas de FR.2

O objetivo deste trabalho é avaliar a utilidade do quadro clínico para o diagnóstico da FAA estreptocócica e definir um agrupamento de sinais e sintomas que maximize o seu poder diagnóstico.

## Método

O presente estudo foi conduzido com pacientes com 18 anos ou menos, que se apresentaram com queixa de dor de garganta e/ou eritema de faringe e/ou amígdalas à admissão, em um serviço de emergência e em um ambulatório de pediatria, na cidade de Belo Horizonte, Minas Gerais, Brasil e foi aprovado pelo comitê de ética da Universidade Federal de São João Del Rey (UFSJ) sob parecer número 05705112.6.0000.5545. Pacientes que haviam utilizado penicilina benzatina nos últimos 30 dias ou qualquer outro agente antimicrobiano nos últimos 15 dias foram excluídos do estudo.

Sinais e sintomas associados com o trato respiratório superior e FAA foram pesquisados e registrados em um formulário padronizado. Além disso, também foram pesquisados sinais e sintomas que não foram frequentemente analisados por estudos similares, como os seguintes: gengivite, lacrimejamento, hiperemia de pilares amigdalianos, espirros e prostração durante e entre os picos febris (>38,5º) . 

Depois de obter consentimento dos responsáveis, foi realizado o exame físico, sendo o teste de aglutinação de partículas do látex (TAPL) e cultura de orofaringe obtidos de forma independente e cega. Dois *swabbs* foram obtidos de cada paciente, um para o TAPL (PathoDx^(r)^, DPC, Los Angeles, USA) e o outro para a cultura convencional em placa de agár-sangue de carneiro a 5%.

Distintamente de quase todos os estudos publicados neste assunto, o protocolo incluiu não apenas a cultura de orofaringe como padrão ouro, mas um padrão de referência melhor e mais robusto, qual seja, a combinação em paralelo dos resultados da cultura de orofaringe e do TAPL. O diagnóstico de certeza de FAA estreptocócica foi dado quando a cultura ou o TAPL foram positivos; de forma oposta, um resultado negativo para os ambos, cultura e TAPL, descartou o diagnóstico de FAA estreptocócica, aumentando, assim, a sensibilidade do padrão. 

Foram calculadas a sensibilidade e a especificidade para cada sinal e sintoma com seus respectivos intervalos de confiança a 95% (IC95%). A razão de verossimilhança positiva (*likelihood ratio* +), a razão de chances (*odds ratio) *e o *p*-valor para cada sinal e sintoma foram também calculados. O teste do qui-quadrado foi utilizado para determinar a significância estatística. 

Sinais e sintomas que haviam mostrando *p*-valores <0,20 foram submetidos à análise multivariada por meio de modelo de regressão logística para desenvolver um escore de predição clínica. A sensibilidade, especificidade e *likelihood ratio +* para cada pontuação nesse escore foram, então, calculadas. 

## Resultados

Um total de 335 pacientes foi considerado elegível, dos quais 56 (16,72%) tiveram resultado positivo para cultura de EBHGA, 71 (21,2%) para o TAPL e 78 (23,4%) para TAPL e/ou cultura. A concordância entre os testes foi de 91,3% com *kappa* de 0,72. A sensibilidade, a especificidade, a *likelihood ratio *+ (LR+), bem como a razão de chances (OR) e os *p*-valores foram calculados para cada característica clínica verificada na entrevista e exame clínicos e estão apresentadas na tabela 1.

**Tabela 1:** Acurácia do quadro clínico para o diagnóstico de FAA estreptocócica

Observa-se que a característica clínica que apresentou a maior sensibilidade para o diagnóstico de FAA por EBHGA foi a hiperemia de amígdalas (96,2%), seguida pela hiperemia de orofaringe (94,7%), pela febre referida pelo responsável (93,6%) e pela ausência de conjuntivite (91,0%). Contudo, todas essas características apresentaram baixa especificidade (10,8%; 18,2%; 14,6% e 19,6%, respectivamente). A característica clínica que apresentou a maior especificidade foi a presença de exantema (94,4%), seguida de gengivite (92,3%), petéquias no palato (83,3%) e temperatura axilar medida no momento da consulta maior que 38,5ºC (82,3%), entretanto todos com baixa sensibilidade (8,9%; 3,9%; 21,8% e 16,4%, respectivamente).

 As características estatisticamente associadas com a FAA por EBHGA (*p<*0,05) foram ausência de coriza (OR=1,80; *p=*0,040); ausência de conjuntivite (OR=2,47; *p=*0,029); hiperemia de orofaringe (OR=3,99; *p=*0,006); exsudato de orofaringe (OR=2,02; *p=*0,011) e hipertrofia de amígdalas (OR=2,60; *p=*0,007). As características que apresentaram as maiores razões de verossimilhança foram exantema (1,50); exsudato de orofaringe (1,40) e petéquias no palato (1,31). Contudo, tanto exantema quanto petéquias no palato apresentaram amplos intervalos de confiança.

As características que entraram na análise de regressão logística foram: febre referida, ausência de coriza, ausência de conjuntivite, hiporexia, hiperemia de orofaringe, exsudato de orofaringe, hiperemia de amígdalas e hipertrofia de amígdalas. A análise foi feita usando o comando de análise gradual (*stepwise).* As seguintes características foram mantidas no modelo pelo programa: ausência de coriza; hiperemia de orofaringe e exsudato de orofaringe (tabela 2). Os resultados da combinação dessas características deram origem a um escore em que cada uma delas corresponde a um ponto, conforme a tabela 3.

**Tabela 2:** Resultado da análise de regressão logística multivariada com modelo gradual

**Tabela 3:** Pontuação e performance do escore clínico composto por ausência de coriza, hiperemia de orofaringe e exsudato de orofaringe para diagnóstico clínico de faringoamigdalite estreptocócica

Observa-se, como é de se esperar, que, quanto maior a pontuação no escore, maior é a especificidade e menor a sensibilidade do quadro clínico. Assim, um escore de 3 pontos, ou seja, a presença das três características (ausência de coriza, exsudato de orofaringe e hiperemia de orofaringe) corresponde a uma sensibilidade de 45,6%, uma especificidade de 74,5% e uma LR+ de 1,79. Essa LR+ é maior do que em qualquer uma das características clínicas isoladamente, correspondendo ao melhor desempenho do quadro clínico em nossos resultados.

## Discussão

Em nosso estudo, a prevalência de EBHGA entre as crianças e adolescentes com FAA foi de 23%, menor do que a estimada pelo trabalho de Shaikh, Leonard e Martin.^11^ Nessa metanálise, a prevalência da reunião dos 14 trabalhos analisados foi de 37%. Contudo, os autores destacam que há grande heterogeneidade entre esses trabalhos e a sua prevalência variou de 17 a 58%. 

Diversos estudos já foram publicados no sentido de elucidar a acurácia do quadro clínico para o diagnóstico etiológico das FAA em crianças com resultados variáveis.^7^^,^ 8 ^,^ 12 ^-^ 15 Rimoin *et al* 16 demonstraram a existência de importante variação na apresentação clínica da FAA em quatro diferentes países, sugerindo ser incorreto extrapolar as conclusões sobre a acurácia do quadro clínico de uma região para outra. Dessa maneir,a destaca-se a importância do presente trabalho, realizado em um país onde a FR e as suas complicações são um grande problema de saúde pública.

As características clínicas estatisticamente associadas com a presença do EBHGA variam entre os estudos^7^^,^ 8 ^,^ 12 ^-^ 15: petéquias no palato; adenopatia cervical anterior; exsudato de orofaringe; odinofagia; hipertrofia de amígdalas; rush escarlatiniforme, febre >38°C; dores musculares; mau hálito; úlceras orais; sintomas gastrintestinais; contato com pacientes com FAA por EBHGA; idade entre 5 e 12 anos e eritema de orofaringe aparecem isolados ou combinados de forma variada nos diversos trabalhos. Coriza, tosse e conjuntivite geralmente aparecem como fatores associados à infecção viral (*p<*0,05). Contudo, nenhuma dessas características é exclusiva da FAA estreptocócica ou viral. Além disso, nenhuma delas apresenta, ao mesmo tempo, alta sensibilidade e especificidade para o diagnóstico da FAA estreptocócica.

Os resultados mostrados em nosso trabalho corroboram essas informações, havendo características com alta sensibilidade, mas baixa especificidade (hiperemia de amígdalas, hiperemia de orofaringe, febre referida e ausência de conjuntivite) e, inversamente, características com alta especificidade, mas baixa sensibilidade (presença de exantema, gengivite, petéquias no palato e temperatura axilar maior que 38,5 °C). A característica com maior equilíbrio entre sensibilidade e especificidade foi o exsudato de orofaringe, com 60% e 57,4%, respectivamente.

Além disso, as características estatisticamente associadas com a presença de EBHGA neste trabalho, quais sejam, ausência de coriza, ausência de conjuntivite, hiperemia de orofaringe, exsudato de orofaringe e hipertrofia de amígdalas, foram diferentes daquelas obtidas em outros estudos realizados no Brasil.^8^^,^ 15 ^,^ 16 Isso reforça ainda mais o achado da variabilidade das manifestações da FAA estreptocócica, mesmo dentro de um mesmo país.

Nenhuma das características analisadas na presente pesquisa apresentou uma *likelihood ratio* elevada. As características com maiores *likelihood ratio* foram: exantema (LR 1,50; IC95% 0,07-29,29); exsudato de orofaringe (LR 1,40; IC95% 1,34-1,48) e petéquias no palato (LR 1,31; IC95% 0,82-2,07). Nota-se, entretanto, que tanto exantema quanto petéquias no palato mostram amplos intervalos de confiança e passam pelo um, o que torna tais resultados imprecisos e pouco significativos. De qualquer modo, qualquer desses valores altera em muito pouco a probabilidade pré-teste da FAA estreptocócica. Se considerarmos uma prevalência média de 30% de EBHGA como causa das FAA^17^, uma *likelihood ratio *positiva de 1,4, como observado para "exsudato de orofaringe", elevaria a probabilidade pós-teste para apenas cerca de 35%. 

Para tentar aumentar a utilidade diagnóstica do quadro clínico, alguns autores têm proposto o uso de escores de predição clínica. Dessa maneira, McIsaac *et al* 6 obtiveram um escore com sensibilidade e especificidade de 66,1% e 85,4%, respectivamente. Contudo, a amostra destes autores é, em sua maior parte, de pacientes adultos, embora seu escore seja corrigido para a idade.

Smeesters *et al* 8 e Joachim, Campos e Smeesters^9^ desenvolveram seus escores direcionados para diagnosticar os casos de FAA não estreptocócica. Eles obtiveram escores com sensibilidade e especificidade para esse fim de 41% e 84% e 35% e 88%, respectivamente. De qualquer maneira, o que se procura com esses escores é oferecer uma alternativa aos testes microbiológicos para o correto manejo das FAA, isto é, oferecer antibióticos adequados quando eles são necessários e evitar o seu uso quando desnecessários, especialmente devido à atual preocupação com o desenvolvimento de resistência bacteriana.^18^

Em nosso estudo, obtivemos sensibilidade de 45,6% (IC95% 34,3-57,3%) e especificidade de 74,5% (IC95% 68,3-79,9%) quando combinaram-se os três sintomas associados com FAA por EBHGA após a análise de regressão logística multivariada. Isso corresponde também a uma *likelihood ratio* de 1,79 (IC95% 1,60-2,00). Como o escore é pouco sensível, ele não permite excluir o diagnóstico de FAA estreptocócica apenas por esse critério. E, apesar da especificidade moderada, o escore de 3 pontos tem uma *likelihood ratio* considerada baixa. Mais uma vez, com o exemplo de uma prevalência de FAA estreptocócica de 30% (probabilidade pré-teste), obter-se-ia uma probabilidade pós-teste de cerca de 40%, que não é suficiente para confirmar o diagnóstico. 

Tendo em vista os importantes impactos da FAA, especialmente a FR e CR, de modo particular nos países menos desenvolvidos, é preciso pensar e definir qual seria a melhor estratégia para o diagnóstico e manejo das FAA, levando em consideração os riscos individuais e coletivos, a disponibilidade dos testes laboratoriais para o diagnóstico, as limitações desses testes e os custos de cada estratégia, sem perder de vista as possíveis complicações de cada uma delas.

Nesse sentido, alguns autores fizeram esforços para definir qual estratégia seria a mais adequada para o manejo das FAA.^19^^-^ 22 Giraldez-Garcia *et al*,^19^ por exemplo, analisaram o custo, a efetividade e a relação custo/efetividade de seis diferentes estratégias para o manejo das FAA na Espanha e concluíram que a melhor estratégia para esse país, em termos de custo e custo/efetividade, seria o uso de escore clínico, seguido de teste rápido para os pacientes com escore elevado, para a confirmação diagnóstica. Ao aplicar essa estratégia à população do presente estudo, seriam testados 30% dos pacientes, o antibiótico de forma inadequada não seria usado em nenhum dos casos negativos para EBHGA, mas 57% dos casos positivos deixariam de ser tratados corretamente, uma porcentagem inaceitável. De qualquer forma, essa extrapolação é apenas um exercício teórico, visto que as informações usadas por esses investigadores podem não se aplicar ao Brasil, no contexto das considerações acima colocadas sobre a variabilidade da FAA entre as diferentes regiões. Além do mais, o critério clínico usado pelos autores espanhóis difere do encontrado neste trabalho. 

Na revisão da literatura, encontrou-se apenas um trabalho que, como o aqui apresentado, tenha usado como padrão de referência a combinação de cultura e de um teste rápido de detecção de antígenos.^9^ Contudo, nesse trabalho, as crianças submetidas à cultura não o foram ao TRIA e vice versa, porque o estudo foi desenvolvido em duas etapas e, em cada uma delas, um dos testes foi usado. No presente estudo, todos os pacientes foram submetidas aos dois testes. Tendo em vista que, na prática, o resultado positivo tanto da cultura quanto do TRIA devem ser encarados como positivos, consideramos que a metodologia aplicada nesse estudo se aproxima mais da realidade. Além disso, o uso de dois testes altamente específicos diminui a possibilidade de resultados falso-negativos, ao aumentar a sensibilidade do padrão.

A presente investigação tem algumas limitações. Em primeiro lugar, dada a inabilidade dos padrões de referência, seja o TAPL, a cultura ou a combinação de ambos, para diferenciar o estado de portador do EBHGA do doente, pode-se ter subestimado a verdadeira acurácia do quadro clínico para o diagnóstico da FAA. Alguns estudos estimam uma prevalência de cerca de 10% de portadores sãos do EBHGA,^11^^,^ 23 contudo não existem métodos precisos e práticos para diferenciar portadores sintomáticos (adoecidos por outro microorganismo que não o EBHGA) dos doentes. Além disso, como o estudo foi transversal, não foi possível avaliar o impacto que avaliações subsequentes poderiam ter na acurácia do quadro clínico. Isso seria importante, já que uma conduta frequente nos casos FAA é aguardar a evolução dentro de 24 a 48 horas para reavaliação das crianças e definição da conduta terapêutica. Não encontramos nenhum trabalho na literatura que tenha assim procedido. Destarte, seria importante, em vista das informações já disponíveis e da contribuição deste trabalho, avançar com a realização de estudos longitudinais, para avaliar de forma sequencial os pacientes e analisar se tal medida contribuiria para aumentar a acurácia diagnóstica do quadro clínico. Além disso, seria interessante proceder a uma análise de custo/efetividade que se aplicasse melhor à realidade brasileira e de outros países em desenvolvimento e subdesenvolvidos. 

Os resultados deste trabalho apontam que o quadro clínico não deve ser usado isoladamente para a confirmação do episódio de FAA estreptocócica. Mesmo quando são combinadas algumas características clínicas, a *likelihood ratio *positiva resultante não permite aumentar a probabilidade pós-teste para um valor suficientemente alto para confirmar o diagnóstico de FAA estreptocócica. É necessário aumentar a disponibilidade de testes laboratoriais confirmatórios, em especial o TRIA, que permite o diagnóstico rápido e acurado do episódio de FAA estreptocócica. 

Em vista da realidade brasileira, em que há a indisponibilidade e a falta de solicitação dos exames laboratoriais confirmatórios, numa retroalimentação contínua, seria importante que houvesse da parte das sociedades afins, como Sociedade Brasileira de Pediatria, Sociedade Brasileira de Infectologia e Sociedade Brasileira de Cardiologia, bem como do Ministério da Saúde, uma recomendação para o correto diagnóstico e manejo das FAA. Dessa forma, haveria um incentivo, tanto aos profissionais médicos, quanto aos laboratórios, para solicitação e disponibilização dos testes necessários para o diagnóstico correto e manejo das FAA.
